# A multidisciplinary active lifestyle aftercare programme for individuals with acquired brain injury (ABI-MOTION): protocol of a practice-based implementation study

**DOI:** 10.1136/bmjopen-2026-118358

**Published:** 2026-07-13

**Authors:** Elisabeth A de Vries, Erik Grauwmeijer, Erwin Ista, Susan JA Blom-De Heer, Jane M Cramm, Gerard M Ribbers, Rita J G van den Berg-Emons, Majanka H Heijenbrok-Kal

**Affiliations:** 1 Erasmus MC, University Medical Center Rotterdam, Department of Rehabilitation Medicine, Rotterdam, The Netherlands; 2 Rijndam Rehabilitation, Rotterdam, The Netherlands; 3 Erasmus MC, University Medical Center Rotterdam, Department of Internal Medicine, Section Nursing Science, Rotterdam, The Netherlands; 4 Erasmus MC-Sophia Children’s Hospital, University Medical Center Rotterdam, Department of Neonatal and Pediatric Intensive Care, Division of Pediatric Intensive Care, Rotterdam, The Netherlands; 5 Department of Socio-Medical Sciences, Erasmus School of Health Policy & Management, Erasmus University Rotterdam, Rotterdam, The Netherlands

**Keywords:** REHABILITATION MEDICINE, Implementation Science, Brain Injuries, Quality of Life, Exercise

## Abstract

**Abstract:**

**Introduction:**

This study aims to enhance aftercare for individuals with acquired brain injury (ABI), focusing on an active lifestyle and health-related quality of life, by implementing a multidisciplinary intervention using a network of outpatient rehabilitation and community-based services, and by evaluating its implementation process and outcomes.

**Methods and analysis:**

The ABI-MOTION intervention includes 5 activities delivered through a network of outpatient rehabilitation and community-based services: outpatient rehabilitation, education flyer, introduction to a sports consultant, maximum of 8 hours of sports consultancy and follow-up by a rehabilitation physician. This hybrid type 2 implementation study focuses on implementation outcomes and uses the RE-AIM (Reach, Effectiveness, Adoption, Implementation and Maintenance) framework for a process evaluation (primary) and effect evaluation (secondary). Sixty individuals with ABI will be followed over time, allowing interim evaluation and optimisation of ABI-MOTION. The first group (control) receives outpatient rehabilitation and follow-up, the intervention group receives all 5 activities of the ABI-MOTION intervention. Data related to the implementation will be collected using field notes, focus groups and semi-structured interviews. To explore the effects of the ABI-MOTION intervention, functional and cognitive tests, self-report questionnaires and home measurements will be conducted just before the intervention and at 3, 6 and 12 months post-discharge.

**Ethics and dissemination:**

The study protocol is approved by the Medical Ethics Committee of Erasmus MC (MEC-2023-0623). The results of the study will be disseminated in peer-reviewed journals, conferences and stakeholder meetings.

**Trial registration number:**

NCT06058351.

**STRENGTHS AND LIMITATIONS OF THIS STUDY**

- The iterative design allows the research team to adjust the acquired brain injury (ABI)-MOTION intervention continuously based on ongoing findings.
- The collection of both quantitative and qualitative data from individuals with ABI and stakeholders from the network provides a comprehensive understanding of the feasibility of the ABI-MOTION intervention in care and aftercare practice.
- The combination of existing care and aftercare increases the likelihood of successful and sustainable implementation of the ABI-MOTION intervention.
- The involvement of researchers in participant recruitment limits the ability to fully observe the course of the implementation process during the recruitment phase of the ABI-MOTION intervention.

## Background

 Acquired brain injury (ABI) refers to any brain damage that occurs after birth.^1^ Stroke and traumatic brain injuries (TBIs) are the most common causes of ABI, with annual global incidence rates of approximately 12 million^2^ for stroke and 69 million^3^ for TBIs. Many people experience long-term consequences of ABI in physical, psychosocial and cognitive functioning.^4 5^ These impairments have a detrimental impact on health-related quality of life (HR-QoL) and participation in society, and may pose challenges for integrating and sustaining a physically active lifestyle after the injury.^6^,^10^

Indeed, individuals with ABI are found to be less physically active than healthy individuals and to have difficulties returning to pre-injury physical activity (PA) levels.^11^,^15^ Also, they often do not adhere to the physical behaviour (PB) recommendations for people with a disability.^11^,^15^ PB can be considered in a 24-hour cycle of PA, sedentary behaviour (SB) and sleep,^16^ with exercise being a specific type of PA, usually performed to improve physical fitness.^17 18^ In the current study, the focus is on integrating and maintaining an active lifestyle by promoting sports and exercise, encouraging everyday PA, such as gardening or walking, and reducing SB. This is particularly important for people with a disability, as it can help alleviate fatigue, anxiety and depression, improve physical fitness, cardiovascular fitness and HR-QoL, and reduce the risk of chronic comorbidities.^19^,^22^

In the Netherlands, community-based services provide guidance to help individuals with ABI adopt an active lifestyle, including consultation to identify suitable sport or exercise opportunities, which has been shown to be effective.^23 24^ However, the connection of these services with clinical rehabilitation or aftercare is not standard practice, creating difficulties for individuals with ABI to locate and access them. Therefore, the overall aim of this study is to improve aftercare for individuals with ABI by promoting an active lifestyle and enhancing HR-QoL. To this end, an intervention (ABI-MOTION) will be implemented using a network of outpatient rehabilitation and community-based services, and its implementation and effects will be evaluated.

## Methods and analysis

### Study design

**Figure 1:**
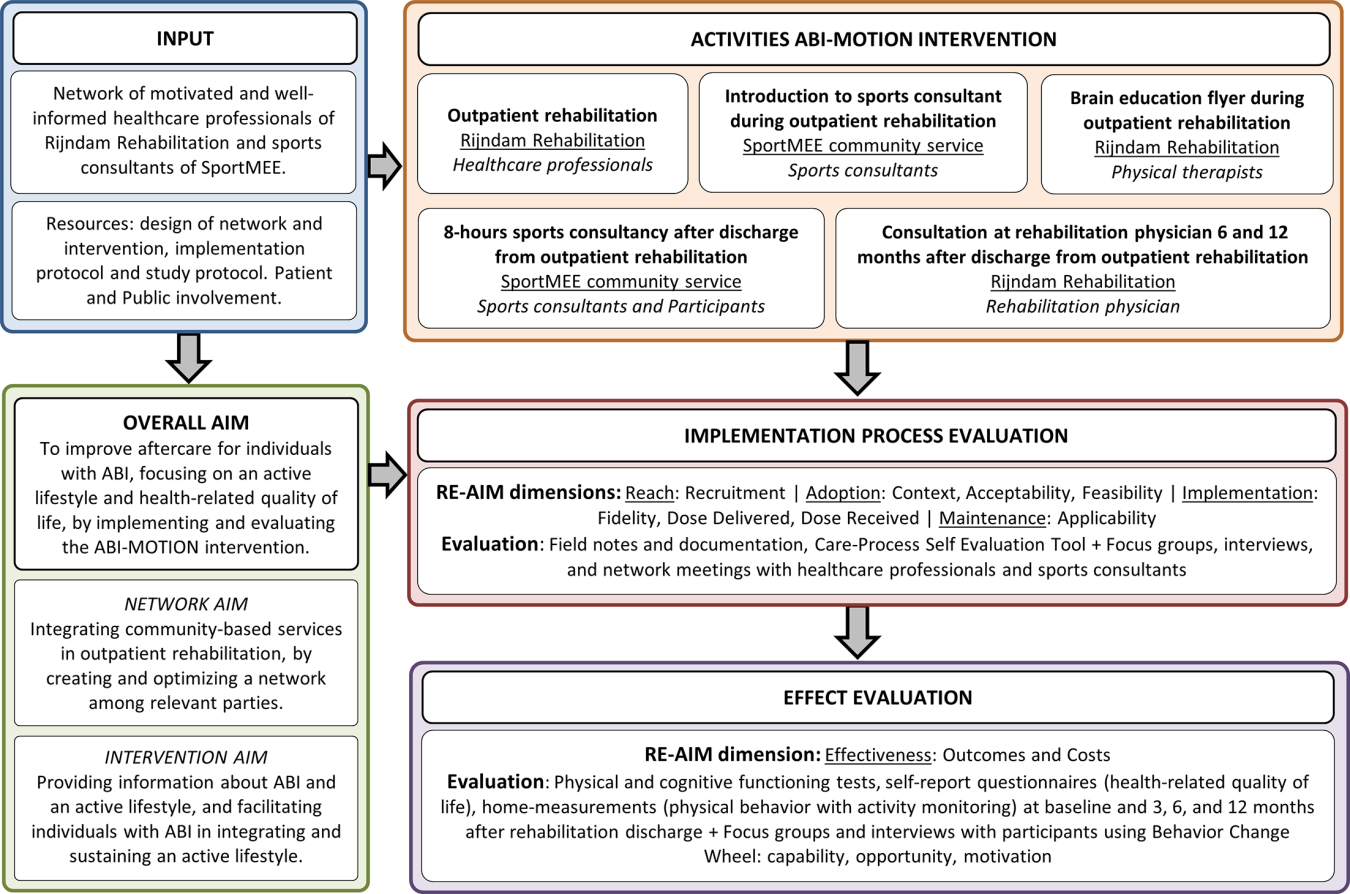
Logic diagram of ABI-MOTION. ABI, acquired brain injury; RE-AIM, Reach, Effectiveness, Adoption, Implementation and Maintenance.

ABI-MOTION is a hybrid type 2 implementation study,^25^ with implementation outcomes as the primary focus. The study includes a process and effect evaluation, using the RE-AIM framework (Reach, Effectiveness, Adoption, Implementation and Maintenance).^26^ The study protocol has been registered on ClinicalTrials.gov (NCT06058351) and approved by the Medical Ethics Committee of Erasmus MC (MEC-2023-0623). A logic diagram of the ABI-MOTION study is presented in figure 1. The logic diagram provides a schematic overview of inputs, aims, and activities of the ABI-MOTION intervention, the implementation process evaluation (primary) and effect evaluation (secondary) parts, and the outcomes and outputs of the study. The start date of the study was 1 March 2024 and the planned end date of the study is 1 March 2027.

### Patient and public involvement

To identify the stakeholders relevant for patient and public involvement in the ABI-MOTION project, we initially engaged the most apparent stakeholders, including individuals with ABI, physical therapists, movement therapists and a community-based service involved in sports and exercise consultancy. Through discussions with these stakeholders, we explored whether any stakeholders were missing. This revealed other relevant stakeholders, such as physicians from the rehabilitation centre, other community-based services involved in sports provision and the association of individuals with brain injuries in the greater Rotterdam region.

The stakeholder group consisted of two individuals with ABI and one partner of an individual with ABI. In addition, we involved a physical therapist, a rehabilitation physician and a movement therapist from the rehabilitation centre. Furthermore, a representative from the association of individuals with brain injuries in the greater Rotterdam region was included, as well as a representative from the community-based service.

Individuals with ABI were involved during the grant application phase, by providing input on the relevance and feasibility of the study. They also contributed to the development of the study protocol, to ensure that the research questions, outcome measures and time investment were in line with their needs, experiences and abilities. During the study, individuals with ABI (ie, study participants) will continue to be involved in optimising the ABI-MOTION intervention through focus groups and semi-structured interviews. At least 4 focus groups and 8 semi-structured interviews will be organised throughout different phases of the study.

To optimise the implementation and study outcomes, an independent user committee has been established. The user committee also includes individuals with ABI, representatives from other rehabilitation centres and professionals from community services in different regions. The role of the committee is to provide independent feedback on the study design, process and outcomes. In addition, the researchers may ask specific questions to the committee for advice. The committee will meet once a year. The insights and recommendations from the user committee sessions will be used for dissemination plans and for drafting a plan for scaling the ABI-MOTION intervention to other regions.

The major changes made as a result of the patient and public involvement to the grant application and subsequent study protocol included the inclusion of a control group prior to the implementation of the ABI-MOTION intervention and the delivery of the sports consultation on an individual basis instead of in a group format. Throughout the study, the ABI-MOTION intervention protocol may be further adapted based on feedback obtained from the focus groups, semi-structured interviews and meetings of the user committee.

### Network

Rijndam Rehabilitation and MEE Rotterdam Rijnmond are the two main network partners. Rijndam Rehabilitation is an inpatient and outpatient rehabilitation provider with 15 locations in the Rotterdam region, which is an urban region in the mid-western part of the Netherlands.^27^ MEE NL is a cooperative association of 20 regional MEE organisations aiming to improve social participation for everyone, including people with a disability.^23^ The sports consultancy programme (SportMEE) is a community-based initiative, funded by the municipality of Rotterdam that aims to advise and guide individuals with disabilities to exercise, sport and an active lifestyle. This sports consultancy is tailored to the patient and may entail guidance to an active lifestyle, support in finding a suitable sport or exercise location, practising with travelling to a sport or exercise location and easily accessible support in the long-term.^24^

Other stakeholders in the network include community-based sports and exercise initiatives in the Rotterdam region, such as those supporting healthcare professionals in finding sports activities for their patients and those engaged in creating new sports provision.

### Study population

The study population will consist of individuals with ABI, healthcare professionals from Rijndam Rehabilitation, including physical and movement therapists and rehabilitation physicians, sports consultants from the community-based service and other stakeholders from the network. Inclusion criteria for individuals with ABI are that they are receiving outpatient rehabilitation at Rijndam Rehabilitation and are at least 18 years of age. Individuals with a life expectancy of less than 1 year or with severe cognitive impairments that preclude providing informed consent and sufficient understanding of study procedures are excluded from the study. Capacity to consent and participate is assessed by the treating rehabilitation physician.

### Power analysis

Since the implementation process is the primary outcome, this study is powered based on the number of individuals with ABI who will receive sports consultancy through the SportMEE programme. Currently, less than 10% of the target population receives sports consultancy via SportMEE. After implementation of the ABI-MOTION intervention, we hypothesise that the reach of the programme will increase to 60%. This means that 60% of participants introduced to the sports consultant during rehabilitation will continue with the optional SportMEE consultancy programme after rehabilitation discharge. Based on these proportions and using an intervention group and a control group with a 3:1 allocation ratio, a two-sided alpha of 0.05 and a power of 0.80, a total sample size of 48 participants is required, 36 in the intervention group and 12 in the control group, as calculated with the Fisher’s exact test. Based on this calculation and taking into account a potential drop-out of 20%, we strive to include a cohort of 60 patients, starting with 15 participants before implementation of the ABI-MOTION intervention, followed by 45 participants in the intervention group after implementation.

### Recruitment process

Consecutive patients with ABI who follow outpatient rehabilitation at Rijndam Rehabilitation are screened for eligibility based on the inclusion and exclusion criteria by one of their treating therapists. Eligible patients receive an invitation letter from their therapist for the ABI-MOTION study and are asked for their consent to be contacted by the researchers. If they agree, the researcher will contact the patient after a reflection period of 1 week. Prior to inclusion, the participant is asked to provide written informed consent for the study. Therapists and physicians from Rijndam Rehabilitation, sports consultants from SportMEE and other network stakeholders will be recruited by one of the researchers.

### ABI-MOTION intervention

The ABI-MOTION intervention consists of 5 activities (figure 1). The programme starts with outpatient rehabilitation for patients with ABI (1), provided in the rehabilitation centre by a multidisciplinary team which may include a rehabilitation physician, physical therapist, occupational therapist, psychologist and/or movement therapist. In addition, during outpatient rehabilitation a brain education flyer is provided by a physical therapist (2), with information about ABI, PB guidelines and benefits of an active lifestyle. The community-based service will be integrated in outpatient rehabilitation by arranging an introduction of the participant to a sports consultant during outpatient rehabilitation (3). After rehabilitation discharge, patients may receive a maximum of 8 hours of sports consultancy, depending on their needs, which is delivered by the sports consultants of the SportMEE programme (4). There are no eligibility criteria for the SportMEE consultancy programme. About 6 and 12 months after rehabilitation discharge, the patient has follow-up consultations with the rehabilitation physician (5) as part of outpatient rehabilitation care. The control group receives activities 1 and 5. The intervention group receives all 5 activities.

### Implementation of ABI-MOTION intervention

An implementation protocol for the ABI-MOTION intervention was developed prior to the start of the study. This protocol describes relevant stakeholders, expected implementation determinants and corresponding implementation strategies. In addition, potential barriers and facilitators for implementation are described separately as more specific determinants of the implementation process.

Key stakeholders in the implementation process include healthcare professionals from Rijndam Rehabilitation, sports consultants from SportMEE and individuals with ABI. In addition, other sports or exercise organisations in the Rotterdam region, as well as the management teams of both Rijndam Rehabilitation and SportMEE are expected to play a role in the implementation of the ABI-MOTION intervention.

Based on prior discussions with the stakeholders during the preparation phase of the study (unpublished), knowledge, awareness and self-efficacy are expected to be important implementation determinants. Table 1 presents an overview of these determinants with the corresponding implementation strategies according to the taxonomy of behaviour change methods.^28^ Ensuring that all stakeholders have sufficient knowledge on the ABI-MOTION intervention and the benefits of an active lifestyle, through discussion and elaboration and improving awareness and self-efficacy of the stakeholders by raising consciousness and guided practice*,* is expected to contribute to successful implementation of the ABI-MOTION intervention. Table 2 describes examples of more specific determinants for implementation of the ABI-MOTION intervention. These barriers and facilitators are categorised according to six levels of healthcare as described by Grol and Wensing.^29^

**Table 1:** Description of the potential important implementation determinants and corresponding strategies

Determinant	Strategy	Description
Knowledge	Discussion	Researchers engage healthcare professionals in structured discussions about the activities of the ABI-MOTION intervention; healthcare professionals discuss the intervention and the benefits of an active lifestyle with individuals with ABI
	Elaboration	Sports consultants emphasise the importance and benefits of an active lifestyle during their contact with the participant; after rehabilitation discharge, physicians and sports consultants continue to repeat and highlight this in follow-up consultations
Awareness	Consciousness raising	Researchers provide information to healthcare professionals and sport consultants about the ABI-MOTION intervention and its added value to standard rehabilitation care; healthcare professionals and sports consultants raise awareness in individuals with ABI about participating in the ABI-MOTION intervention and the benefits of an active lifestyle during and after rehabilitation
Self-efficacy	Guided practice	Researchers provide feedback on the implementation of the ABI-MOTION intervention to healthcare professionals and sports consultants; individuals with ABI are supported by healthcare professionals and sports consultants with feedback and encouragement to integrate physical activity into daily life in the long term

ABI, acquired brain injury.

**Table 2:** Level of healthcare and related potential barriers and facilitators

Level	Barriers (B) and facilitators (F)
Innovation	B: Feasibility of the integration of SportMEE in Rijndam F: Positive experiences with ABI-MOTION intervention activities
Individual health professional	B: Existing routines of the healthcare professionals F: Interest of healthcare professionals in ABI-MOTION
Patient	B: Lack of knowledge, not aware of advantages of an active lifestyle F: Rehabilitation phase ‘window of opportunity’
Professional interactions	B: Communication between rehabilitation and SportMEE F: One contact person of SportMEE
Organisation	B: Capacity SportMEE, planning of care F: No extra or new care, but combination of existing (after)care
Society	B: Costs of (after)care, limited financial resources F: Experience SportMEE in Rotterdam region (cultural context)

ABI, acquired brain injury.

Prior to the implementation of the ABI-MOTION intervention and during the recruitment period of the control group (ie, phase I), the implementation protocol, including the roles of all stakeholders, will be specified and described in more detail. In addition, all study procedures will be discussed with all stakeholders. After finishing inclusion of the control group, the ABI-MOTION intervention will be implemented and inclusion of the intervention group will start. During this second phase, the implementation protocol will be applied and optimised by verifying the implementation determinants described in tables1  2. The corresponding implementation strategies are also verified and may be updated if needed, according to the five steps of the implementation mapping method: (1) analysis of implementation factors, (2) defining change goals, (3) choosing useful theories and congruent strategies, (4) selecting strategies and (5) practical plan for a tailored implementation strategy.^28 30^

### Data collection

For the process evaluation, participants, healthcare professionals and sport consultants are asked to participate in 1 or 2 focus groups and/or semi-structured interviews in different phases of the study. The focus groups and semi-structured interviews are conducted by 2 researchers according to a predefined topic list. In addition, network meetings will be organised at least once a year, with the researchers and network partners. Furthermore, the researchers will document process outputs throughout the whole study. Focus groups and semi-structured interviews will be recorded and transcribed verbatim. The network meetings will not be recorded, but the discussions will be documented directly afterwards and shared with all stakeholders for their additional input. Outputs from the focus groups, interviews and network meetings will be analysed directly after the meetings and will be used to adjust and optimise the ABI-MOTION intervention and its implementation.

For the explorative effect evaluation, participants are followed up to 1 year after discharge from outpatient rehabilitation and will undergo four measurements; a baseline measurement within the last 4 weeks of outpatient rehabilitation and before the start of the other parts of the ABI-MOTION intervention (T0), and at 3 (T1), 6 (T2) and 12 (T3) months post-rehabilitation discharge. Measurements include physical and cognitive functioning tests at the rehabilitation centre, home measurements including activity monitoring and an electronic diary, and (online) self-report questionnaires sent to the participants by mail or postal mail. The T1 measurement consists of (online) self-report questionnaires only. The T0 measurement aligns with an outpatient rehabilitation visit, and T2 and T3 with standard care follow-up visits at the rehabilitation physician (figure 1). During the visits at the rehabilitation centre (T0, T2 and T3), a trained research assistant conducts the tests, asks general questions and provides information and requirements for the home measurements. To reduce potential burden, participants are given flexibility to complete the questionnaires at their own pace, including the option to pause and resume at a later time. Since implementation outcomes are the primary focus, participants who are unable to complete the questionnaires independently (eg, due to aphasia) are not required to do so. Support of proxies in completing the questionnaires is allowed; however, proxies are asked not to complete the questionnaires on behalf of participants.

## Evaluation

The evaluation of the ABI-MOTION intervention will be conducted using the RE-AIM framework, which provides a comprehensive structure for assessing both the implementation process and effects of the ABI-MOTION intervention. Table 3 presents a description of the dimensions of the RE-AIM framework and corresponding evaluation outcomes.

**Table 3:** The RE-AIM framework and corresponding evaluation outcomes for the ABI-MOTION intervention

Dimension	Subtheme	Main question	Evaluation outcomes
Reach	Recruitment	Did the intervention reach the intended population?	Number of patients with ABI in outpatient rehabilitation; potential participants; invited, included and excluded participants and reasons for non-inclusion
Effectiveness	Outcomes Costs	What are the effects and the costs of the intervention?	See table 4 for all effect outcomes
Adoption	Context Acceptability Feasibility	Which contextual factors have influenced the implementation and to what extent was the ABI-MOTION intervention perceived as acceptable and feasible by the stakeholders?	Contextual factors, acceptability, feasibility and barriers and facilitators for implementation of all stakeholders.
Implementation	Fidelity Dose delivered Dose received	To what extent was the ABI-MOTION intervention implemented as intended?	Protocol adherence for the new activities of the ABI-MOTION intervention
Maintenance	Applicability	Can ABI-MOTION be sustained and integrated into routine care in the Rotterdam region?	Care Process Self-Evaluation Tool questionnaire for healthcare professionals; barriers and facilitators for sustained delivery of the ABI-MOTION intervention, especially after the inclusion and research phase

ABI, acquired brain injury; RE-AIM, Reach, Effectiveness, Adoption, Implementation and Maintenance.

**Table 4:** Outcomes effect evaluation

	During outpatient rehabilitation	T0: baseline before discharge and start of intervention	T1: 3 months post discharge	T2: 6 months post discharge	T3: 12 months post discharge
Physical and cognitive functioning test at the rehabilitation centre					
1 min sit-to-stand test		X		X	X
10 m walk test		X		X	X
Handgrip strength: JAMAR dynamometer		X		X	X
Cognitive functioning: MoCA		X		X	X
Aphasia: ABC		X		X	X
Functional ambulation categories		X		X	X
Anthropometry		X		X	X

Self-report questionnaires: online (or postal) at home					
Participant characteristics		X	X	X	X
Clinical characteristics		EPR	X	X	X
General questions		X	X	X	X
Physical activity: IPAQ		X	X	X	X
Physical fitness: IFIS		X	X	X	X
Anxiety/depression: HADS		X	X	X	X
Cognitive complaints: CFQ		X	X	X	X
Quality of life: EQ-5D-5L, SF-36		X	X	X	X
Coping: CISS-SF		X	X	X	X
Fatigue: CIS		X	X	X	X
Participation: USER-P		X	X	X	X
Healthcare use: iMCQ		X	X	X	X
Return to work: iPCQ		X	X	X	X

Home measurements					
Physical behaviour: GENEActiv		X		X	X
Intention/motivation: electronic diary		X		X	X

Focus groups and Interviews					
BCW: capability, opportunity, motivation	X	X	X	X	X

ABC, Aphasia Bedside Check; BCW, Behavior Change Wheel; CFQ, Cognitive Failure Questionnaire; CIS, Checklist Individual Strength; CISS-SF, Coping Inventory for Stressful Situations – Short Form; EPR, Electronic Patient Record; EQ-5D, EuroQoL-5D; HADS, Hospital Anxiety and Depression Scale; IFIS, International FItness Scale; iMCQ, iMTA Medical Consumption Questionnaire; IPAQ, International Physical Activity Questionnaire; iPCQ, iMTA Productivity Cost Questionnaire; MoCA, Montreal Cognitive Assessment; SF-36, Short Form Health Survey-36; USER-P, Utrecht Scale for Evaluation of Rehabilitation – Participation; X, study measurement.

### Evaluation of implementation process

The evaluation of the implementation process addresses the RE-AIM dimensions: reach, adoption, implementation and maintenance (table 3). These dimensions will be explored through both quantitative and qualitative methods, including documentation, registration, focus groups, semi-structured interviews and network meetings.

Reach will be assessed by quantifying the extent to which the intervention successfully reached the target population (ie, individuals with ABI who follow outpatient rehabilitation). This will be expressed in the total number of patients in outpatient rehabilitation, the number of potential participants identified and those invited, included and excluded from the study. In addition, reasons for non-inclusion will be systematically documented to gain insight into recruitment barriers and representativeness of the study population.

Regarding adoption, the focus will be on the contextual factors that influence the uptake of the intervention, as well as the perceived acceptability and feasibility among all stakeholders. In assessing these aspects, the focus will be on whether the ABI-MOTION aligns with existing care processes, adoption of ABI-MOTION at the organisational level and stakeholder experiences throughout the process of optimising the ABI-MOTION intervention.

The implementation dimension will be expressed in the extent to which the ABI-MOTION protocol was adhered to in practice. This includes measures of dose delivered and dose received, for example, number of introductions to sports consultant during outpatient rehabilitation. These kinds of protocol adherence and deviation measures will be documented and described.

Finally, maintenance will be evaluated using the Care Process Self-Evaluation Tool (CPSET)^31^ filled in by healthcare professionals. In addition, barriers and facilitators for sustained delivery of the ABI-MOTION intervention after the inclusion period and research phase will be explored.

### Evaluation of intervention effects

The effect evaluation focuses on the effectiveness dimension of the RE-AIM framework, by exploring the effects of the ABI-MOTION intervention on several outcomes and associated costs. An overview of all outcomes for the effect evaluation is provided in table 4. The focus is on objectively assessed PB and HR-QoL. PB will be assessed for 7 consecutive days with the GENEActiv (Activinsights, Kimbolton, England), which is a validated wrist-worn accelerometer.^32^ PB will be expressed in PA and SB, including total minutes of PA, light PA, moderate to vigorous PA, SB and sleep per day. HR-QoL will be assessed with the EQ-5D-5L^33^ questionnaire and the SF-36.^34 35^ The EQ-5D-5L consists of the 5-item EQ-5D index (mobility, self-care, usual activities, pain/discomfort and anxiety/depression) and a visual analogue scale.^33^ The SF-36 questionnaire assesses limitations in functioning and entails 8 subdomains, which can be combined into a physical and a mental component score.^34 35^

Other outcome measures include the physical and cognitive functioning tests, the self-report questionnaires, the outcomes of the questions of the electronic diary and the patient and clinical characteristics (table 4). The physical functioning tests include the 1 min sit-to-stand test,^36^ 10 m walk test^37^ and hand grip strength test (JAMAR, Lafayette Instrument Company, Lafayette, Indiana, USA),^38^ which are conducted as a measure of aerobic capacity, walking speed and overall muscle strength, respectively. Cognitive functioning is measured with the Montreal Cognitive Assessment^39^ and aphasia, if indicated, with the Aphasia Bedside Check test.^40^ The level of independence in walking is assessed with the Functional Ambulation Categories.^41^ Anthropometry includes length and weight, waist circumference and arm circumference; length and weight, which will be used to calculate body mass index (BMI).

Participant characteristics that will be collected include age, sex, education, living situation (alone/with others), pre-injury work status (paid/unpaid/retired), migration background, BMI, smoking, pre-injury activity status using the Saltin-Grimby Physical Activity Level Scale^42^ and socio-economic status (based on postal code). Clinical characteristics will be collected from the electronic patient file and/or the participant will be asked. Characteristics include type of brain injury (eg, traumatic, stroke), time after injury, time between hospital discharge and start of rehabilitation, type of ABI treatment (eg, surgical, endovascular, conservative and pharmaceutical), length of stay in hospital, intensive care (yes/no), duration of inpatient and outpatient rehabilitation, complications, comorbidities (eg, obesity, cardiovascular disease, pulmonary disease, diabetes and other), and smoking, alcohol and/or drugs use.

Validated self-report questionnaires are used to assess, PA (International Physical Activity Questionnaire),^43^ physical fitness (International FItness Scale),^44^ symptoms of anxiety and depression (Hospital Anxiety and Depression Scale),^45^ cognitive complaints (Cognitive Failure Questionnaire),^46^ coping (Coping Inventory for Stressful Situations – Short Form),^47^ fatigue (Checklist Individual Strength),^48^ participation (Utrecht Scale for Evaluation of Rehabilitation – Participation),^49^ healthcare use (Institute for Medical Technology Assessment (iMTA) Medical Consumption Questionnaire (iMCQ))^50^ and return to work (iMTA Productivity Cost Questionnaire (iPCQ)).^51^ Data from the iMCQ and iPCQ questionnaires will be used to conduct a descriptive cost analysis focusing on healthcare use and productivity losses. Questions on the electronic diary refer to sleep quality, and intention and motivation for being physically active.

The effects of the ABI-MOTION intervention in terms of incorporating and maintaining an active lifestyle and behaviour change will also be evaluated through focus groups and semi-structured interviews, using the Behavior Change Wheel (BCW)^52 53^ as framework and focusing on the three main components of the BCW, capability, opportunity and motivation.

### Statistical and qualitative analyses

Descriptive statistics will be used to summarise the documented numbers related to the evaluation of the implementation process and the outcomes of the effect evaluation. Means and SD will be calculated for variables on an interval scale, and medians and IQRs for ordinal variables and if variables are not normally distributed. Proportions will be calculated for nominal variables. ATLAS.ti software (V.24, ATLAS.ti Scientific Software Development GmbH, Berlin, Germany) will be used to analyse and interpret the qualitative data of the focus groups and network meetings. The dimensions and subthemes of the RE-AIM framework, and the BCW, will be used to thematically identify and describe the themes that are discussed.

The changes over time (T0–T3) of the effect outcomes will be analysed with Generalised Estimating Equations (GEE) models for repeated measurements on an intention-to-treat basis using SPSS (V.32, IBM SPSS Statistics, New York, USA). This method takes all observations into account, even if some outcomes are missing, assuming that these data are missing at random. Each parameter at each time point will be included as the dependent variable in separate GEE models. Independent variables that will be added to each model include measurement time (T0, T1, T2 and T3), group (intervention vs control), and the interaction between time and group. In post-hoc analyses, differences between the intervention and control group at each time point will be analysed. If differences in participant or clinical characteristics between the control and intervention group are present, the models will be adjusted for patient characteristics (eg, age, sex and living status) or clinical characteristics (eg, type of injury, type of treatment and comorbidity). Maximal number of covariates in the model will be based on the number of participants in both groups. Potential differences in participant and clinical characteristics between the control group and intervention group will be calculated using independent t-tests for normally distributed interval scale variables, the Mann-Whitney U test for non-normally distributed interval scale variables, and the χ^2^ test for categorical variables. Interim analyses will be performed when all patients from the control group or intervention group have finished the baseline, 3 and/or 6 months follow-up visits.

## Discussion

Individuals with ABI have difficulties in integrating and maintaining an active lifestyle after rehabilitation discharge. In line with this, they have expressed a need for long-term (after)care close to home, including guidance on an active lifestyle. This study addresses these aspects by implementing the ABI-MOTION intervention using a network of rehabilitation and community-based services. The hybrid implementation study design provides insight into both the implementation process and intervention effects. This helps to understand which aspects of the intervention work and are feasible in practice.

### Potential benefits

We expect that, after the implementation of the ABI-MOTION intervention, more patients will be guided to an active lifestyle after rehabilitation discharge. If they succeed in adopting and sustaining an active lifestyle, patients may directly benefit from the ABI-MOTION intervention. In addition, since the ABI-MOTION intervention addresses the specific needs of individuals with ABI with regard to the aftercare phase, we expect that their HR-QoL may improve.

### Limitations

This protocol has several limitations. First, as this is a hybrid implementation study with a primary focus on implementation outcomes, the pre–post effect evaluation is considered secondary. Consequently, findings should be interpreted as exploratory. In addition, the involvement of researchers in participant recruitment for the effect evaluation limits the ability to fully observe the course of the implementation process and may influence implementation outcomes. Furthermore, the relatively small sample size limits the number of covariates that can be included in the effect evaluation analyses and may reduce the precision of the estimated effect. Finally, as the study is conducted within a specific regional network in the Netherlands, the generalisability of the findings to other settings or healthcare systems may be limited.

### Ethics, safety and dissemination

This study has been approved by the Medical Ethics Committee of Erasmus MC (MEC-2023-0623). Participation is voluntary and participants can withdraw their consent at any time. This will have no impact on their treatment. The rehabilitation physician examines contraindications for moderate to vigorous exercise in a physical examination, which is standard practice during outpatient rehabilitation. Suffering from contraindications for performing moderate to vigorous exercise and activities is not an exclusion criterion for the study. Instead, the contraindication and specific advice on how to exercise safely will be discussed with the sports consultant. Harms are defined as any adverse events or unintended negative consequences potentially related to study participation. Given the low-risk implementation design, harms will be assessed non-systematically through reporting by the researchers.

The handling of personal data complies with the European Union General Data Protection Regulation and the Dutch Act on Implementation of the General Data Protection Regulation. All data is coded before storing and is therefore not retraceable to the individual patient. The key to the code will be safeguarded by the investigator. Individual de-identified participant data and statistical code will be available after study completion on reasonable request.

The procedures in this study will continuously be monitored by the members of the research team, especially by the investigator. Also, a qualified independent monitor is appointed, who is not a part of the research team. The database will be monitored every 3 months for completeness and accuracy of the data registration. A random check will be performed every quarter of a year on the completeness and accuracy of the data of at least 10 patients. If any, all protocol amendments will be notified to the Medical Ethics Committee of Erasmus MC

The sponsor/investigator has a liability insurance which is in accordance with the legal requirements in the Netherlands (Article 7 Wet medisch-wetenschappelijk onderzoek met mensen). This insurance provides cover for damage to research subjects through injury or death caused by the study. The insurance applies to the damage that becomes apparent during the study or within 4 years after the end of the study.

The results of the study will be disseminated in peer-reviewed journals, conferences and in stakeholder meetings.
