# Supplementary material for: A multidisciplinary active lifestyle aftercare programme for individuals with acquired brain injury (ABI-MOTION): protocol of a practice-based implementation study
Source: BMJ Open. 2026 Jul 13;16(7):e118358. doi: 10.1136/bmjopen-2026-118358 (PMC13365756; doi:10.1136/bmjopen-2026-118358)
Supplement: Supplementary file 1 [file bmjopen-2026-118358.reviewer_comments.pdf]

PEER REVIEW HISTORY

BMJ Open publishes all reviews undertaken for accepted manuscripts. Reviewers are asked to complete a checklist review form and are provided with free text boxes to elaborate on their assessment. These free text comments are reproduced below.

ARTICLE DETAILS

Title (Provisional)

A Multidisciplinary Active Lifestyle Aftercare Program for Individuals with Acquired Brain Injury (ABI-MOTION): Protocol of a Practice-Based Implementation Study

Authors

de Vries, Elisabeth A; Grauwmeijer, Erik; Ista, Erwin; Blom-De Heer, Susan JA; Cramm, Jane M.; Ribbers, Gerard M.; van den Berg-Emons, Rita J.G.; Heijenbrok-Kal, Majanka H.

VERSION 1 - REVIEW

**Reviewer**	**1**
**Name**	**Nair, Krishnan**
**Affiliation**	**The University of Sheffield**
**Date**	**30-Apr-2026**
**COI**	**None**

Thank you very much for sharing your protocol for this novel study to promote active life style among people with ABI. This research will, hopefully facilitate adoption of a healthy life style by the ABI survivors.

I appreciate that authors have had discussions with key stake holders about their intervention. The paper will be strengthened by expanding the patient public involvement with more details

How did you identify the stakeholders?

Details of the stake holders like how many individuals with ABI, how many sports trainers etc?

How many semi structured interviews and how many focus groups?

How did you analyse the output from interviews and focus groups?

What are the major changes made as a result of PPI

The paper will also benefit from a description of limitations of this protocol

Reviewer **2**

Name **Singer , Barbara**

Affiliation **School of Medical and Health Sciences, Edith Cowan University, Joondalup, Australia., School of Medical and Health Sciences**

Date **18-May-2026**

COI **None**

A Multidisciplinary Active Lifestyle Aftercare Program for Individuals with Acquired Brain Injury (ABI-MOTION): Protocol of a Practice-Based Implementation Study

bmjopen-2026-118358

General comments
The topic of this study is important and could lead to improved physical / cognitive function and quality of life for individuals with ABI receiving after care through these service providers, as well as reducing the development of comorbidities associated with inactivity (among other causes). There is potential for the study approach, if proven successful, to be translated to other similar settings. I commend the researchers for their dedication to this important goal, and for considering issues to do with effective implementation and sustainability of the ABI-MOTION program.

Please note, all page numbers referenced in the following comments relate to the number at *the bottom right of each page* in the pdf provided.

I have two main areas of concern
The researchers seem a little unclear whether they are primarily interested in *implementation* of an intervention *that has known efficacy*, or if they want to *explore the efficacy* of ABI-MOTION (with a process evaluation to collect data related to implementation strategies and their effectiveness). This becomes most apparent in the statistical plan provided. I have elaborated these concerns below.

There are a large number of measures for the so called 'effect evaluation', but none are identified as a 'primary outcome variable' (from which a sample size estimation could be undertaken). Instead, the section on 'power analysis' (top of page 7) states that:

"This study is powered based on the number of individuals with ABI who will receive sports consultancy through the SportMEE program. Currently, less than 10% of the target population receives sports consultancy via SportMEE. After implementation of the ABI-MOTION intervention, we hypothesize that the reach of the program increases to 60%".
This is an implementation goal and cannot be used to detect the appropriate sample size appropriate for the proposed study.

Outcome measures ('effect evaluation').

There are a large number of outcome measures, particularly the self report tools. The researchers state that people with lived experience were consulted regarding the appropriateness of the assessments, but this is a very burdensome suite of measures. (As a side note, patient involvement in development of the study protocol is not captured in Figure 1). On P6, (line 46) there is mention 'being mentally incapacitated' as an exclusion criteria. Not only is this inappropriate language, but it is too imprecise. Many people will have some degree of cognitive and/or language impairment post ABI. This very frequently includes issues with memory and attention, which may impact the veracity of self report data. There is a validated assessment for aphasia listed in Table 4, but it is not clear what purpose this will be used for. To exclude potential participants with more severe deficits who will be unable to complete the many self report questionnaires? Will support be provided to assist with completion of self report measures? Will proxy data be allowed (measures completed by a carer)? How will the researchers manage missing data? From my understanding, SPSS takes out the whole case if there are any missing data points (repeated measures).
On P12 it is suggested that costs will be evaluated but no economic analysis is provided. How will these data be derived?

Sample size estimation and statistical analyses
As mentioned above, the power calculation (P7) is based on the premise that the researchers are looking at a simple difference in proportions of participants engaging in the sports consultancy through the SportMEE program. This is an implementation outcome.
However, in the statistical plan (P17) the authors report intending to conduct a repeated measures analysis with four timepoints including the baseline. This is an efficacy outcome. The study is under powered to include the many potential covariates. The authors do not specify what specific covariates will be included in the models, nor what is the justification for their inclusion (ie. what change would they expect to see in the experimental group versus controls). It is recommended that the researchers consult a statistician for the power calculation and the analysis. At the very least, the power calculation (sample size) should be based on the analysis of the primary outcome, and this needs to be determined (what outcome measure will best detect a difference between groups and what is the MDC or MCID of that measure).

Some specific comments re wording / language are listed below for the authors consideration. Wording which could be altered is highlighted in **bold**

P3 line 6. This dot point could be reworded ..."allows **the research team** to adjust..."
P4 line 44. .."shown **to be** effective"..
P5 line 33 ...were in line"... (not where)
P5 line 35 ..."**will** continue to be involved"...
P5 line 42 ..."independent user committee **has been**..."
P5 line 50-51 ...The committee **will meet** once a year.
P6 line 17 ..."aims to advise"...(not advice)
P6 Line 46. A more appropriate term would be those with moderate to severe cognitive impairment (to replace mentally incapacitated).

Recruitment (Page 7) How will capacity to consent be assessed?
P7 lines 23-26 and 46-51 include the same information.
P8 line 32. What are the eligibility criteria for this service (eg. mild to moderate ABI)?
P8 line 48 Should mention be made of carers? Many individuals with ABI will need considerable support (from family/relevant others) to engage in this program.
Table 2, P10, line 11. ..."not aware of the advantages **of an** active lifelstyle"...
P11 Data collection. Lines 12 and 14. Two sentences commence with "In addition..."
P13 line 41. How does 'dose delivered' differ from 'dose received'
P15 line 17. ..."drug us**e**"...

VERSION 1 - AUTHOR RESPONSE

Reviewer 1: Dr. Krishnan Nair, The University of Sheffield

Comments to the Author: Thank you very much for sharing your protocol for this novel study to promote active life style among people with ABI. This research will, hopefully facilitate adoption of a healthy life style by the ABI survivors.

Response: Thank you for reviewing the manuscript, below please find a point-by-point response.

- I appreciate that authors have had discussions with key stake holders about their intervention. The paper will be strengthened by expanding the patient public involvement with more details:
  - How did you identify the stakeholders?;
    Response: We have added the following text to the manuscript at <u>page 5, lines 14-20</u>: "To identify the stakeholders relevant for patient and public involvement in the ABI-MOTION project, we initially engaged the most apparent stakeholders, including individuals with ABI, physical therapists, movement therapists, and a community-based service involved in sports and exercise consultancy. Through discussions with these stakeholders, we explored whether any stakeholders were missing. This revealed other relevant stakeholders, such as physicians from the rehabilitation center, other community-based services involved in sports provision and the association of individuals with brain injuries in the greater Rotterdam region."

  - Details of the stake holders like how many individuals with ABI, how many sports trainers etc?
    Response: We have added the following text to the manuscript at <u>page 5-6, lines 21-23, 1-2</u>: "
    "The stakeholder group consisted of two individuals with ABI and one partner of an individual with ABI. In addition, we involved a physical therapist, a rehabilitation physician, and a movement therapist from the rehabilitation center. Furthermore, a representative from the

association of individuals with brain injuries in the greater Rotterdam region was included, as well as a representative from the community-based service."

- How many semi structured interviews and how many focus groups?
  Response: We have added the following text to the manuscript at <u>page 6, lines 8-9</u>: "
  "At least 4 focus groups and 8 semi-structured interviews will be organized throughout different phases of the study."
- How did you analyse the output from interviews and focus groups?
  Response: During the preparation phase, patient and public involvement consisted of discussions with the stakeholders in various groups. Input from these discussions was directly incorporated into further development of the intervention and study protocol.

  The analysis of the output from the interviews and focus groups during the study phase is described on page 18 of the manuscript: "ATLAS.ti software (version 24) will be used to analyze and interpret the qualitative data of the focus groups and network meetings. The dimensions and subthemes of the RE-AIM framework, and the Behavior Change Wheel, will be used to thematically identify and describe the themes that are discussed."

- What are the major changes made as a result of PPI?
  Response: We have added the following text to the manuscript on <u>page 6, lines 17-22</u>: "The major changes made as a result of PPI to the grant application and subsequent study protocol included the inclusion of a control group prior to the implementation of the ABI-MOTION intervention and the delivery of the sports consultation on an individual basis instead of in a group format. Throughout the study, the ABI-MOTION intervention protocol may be further adapted based on feedback obtained from the focus groups, semi-structured interviews, and meetings of the user committee."

- The paper will also benefit from a description of limitations of this protocol.
  Response: We have added the following text the manuscript on <u>page 19-20, lines 19-24, 1-4</u>:
  "This protocol has several limitations. First, as this is a hybrid implementation study with a primary focus on implementation outcomes, the effect evaluation is considered secondary. Consequently, findings should be interpreted as exploratory. In addition, the involvement of researchers in participant recruitment for the effect evaluation limits the ability to fully observe the course of the implementation process and may influence implementation outcomes. Furthermore, the relatively small sample size limits the number of covariates that can be included

in the effect-evaluation analyses and may reduce the precision of the estimated effect. Finally, as the study is conducted within a specific regional network in the Netherlands, the generalizability of the findings to other settings or healthcare systems may be limited."

Reviewer 2: Dr. Barbara Singer, School of Medical and Health Sciences, Edith Cowan University, Joondalup, Australia.

- **General comments:**

  The topic of this study is important and could lead to improved physical / cognitive function and quality of life for individuals with ABI receiving after care through these service providers, as well as reducing the development of comorbidities associated with inactivity (among other causes). There is potential for the study approach, if proven successful, to be translated to other similar settings. I commend the researchers for their dedication to this important goal, and for considering issues to do with effective implementation and sustainability of the ABI-MOTION program. Please note, all page numbers referenced in the following comments relate to the number at the bottom right of each page in the pdf provided.

  Response: Thank you for reviewing the manuscript, below please find a point-by-point response.

  <u>I have two main areas of concern:</u>

  The researchers seem a little unclear whether they are primarily interested in implementation of an intervention that has known efficacy, or if they want to explore the efficacy of ABI-MOTION (with a process evaluation to collect data related to implementation strategies and their effectiveness). This becomes most apparent in the statistical plan provided. I have elaborated these concerns below. There are a large number of measures for the so called 'effect evaluation', but none are identified as a 'primary outcome variable' (from which a sample size estimation could be undertaken). Instead, the section on 'power analysis' (top of page 7) states that: "This study is powered based on the number of individuals with ABI who will receive sports consultancy through the SportMEE program. Currently, less than 10% of the target population receives sports consultancy via SportMEE. After implementation of the ABI-MOTION intervention, we hypothesize that the reach of the program increases to 60%". This is an implementation goal and cannot be used to detect the appropriate sample size appropriate for the proposed study.

Response: Thank you for addressing these important topics. We agree that the distinction between implementation and effectiveness objectives requires clarification. We have revised the manuscript to explicitly state that this study is a hybrid type 2 implementation study, with implementation outcomes as the primary focus (page 5, lines 3-4 and throughout the manuscript). Effect outcomes (e.g., physical activity and health-related quality of life) are included as secondary and as such exploratory outcomes, consistent with the protocol for the ethical committee and the online trial registry (please see comment editor on page 2 above).

In response to the comment regarding the primary outcome, we agree with the reviewer that the previous version did not sufficiently specify the primary outcome in this hybrid type 2 implementation study. We have clarified that the primary outcome of the study is reach; the percentage of participants introduced to the sports consultant during rehabilitation that continue with the optional SportMEE consultancy program after rehabilitation discharge. With this clarification, we consider the power calculation as appropriate based for this study design. Effect outcomes are analyzed in an exploratory manner, which is now also clarified explicitly in the manuscript.

- **Outcome measures ('effect evaluation').**
  There are a large number of outcome measures, particularly the self report tools. The researchers state that people with lived experience were consulted regarding the appropriateness of the assessments, but this is a very burdensome suite of measures. (As a side note, patient involvement in development of the study protocol is not captured in Figure 1). On P6, (line 46) there is mention 'being mentally incapacitated' as an exclusion criteria. Not only is this inappropriate language, but it is too imprecise. Many people will have some degree of cognitive and/or language impairment post ABI. This very frequently includes issues with memory and attention, which may impact the veracity of self report data. There is a validated assessment for aphasia listed in Table 4, but it is not clear what purpose this will be used for. To exclude potential participants with more severe deficits who will be unable to complete the many self report questionnaires? Will support be provided to assist with completion of self report measures? Will proxy data be allowed (measures completed by a carer)? How will the researchers manage missing data? From my understanding, SPSS takes out the whole case if there are any missing data points (repeated measures). On P12 it is suggested that costs will be evaluated but no economic analysis is provided. How will these data be derived?
  Response: We thank the reviewer for the comments, below we address each point.

- ***Number and burden of outcomes measures****: We acknowledge that the number of outcome measures is extensive and may be burdensome for participants. To reduce this burden, participants are given flexibility to complete questionnaires at their own pace, including the option to pause and resume at a later time. We have added this to the manuscript on page 12, lines 22-24. In addition, previous studies in our department in people with ABI and chronic conditions such as long-COVID have successfully used similar or even larger sets of questionnaires. Finally, patient and public involvement did not identify the number of questionnaires or assessments as a major concern. Therefore we think the number of self-report questionnaires can be considered acceptable and feasible within the context of the study population.*
- ***Figure 1 PPI****: We have added patient and public involvement in figure 1 in the "Input" box, under resources.*
- ***Exclusion criteria: 'being mentally incapacitated' and veracity of self-report data:*** *First, we have changed the term being mentally incapacitated, in line with the response to the editor on page 7, lines 20-22: "Individuals with a life expectancy of less than 1 year or with severe cognitive impairments that preclude providing informed consent and sufficient understanding of study procedures are excluded from the study. Capacity to consent and participate is assessed by the treating rehabilitation physician."*

  Second, we acknowledge that individuals with ABI often have some degree of cognitive and/or language impairment, which may affect the veracity of self-report data. However, many of the questionnaires used in our study have been validated in individuals with ABI. Therefore we consider the outcomes as valid and reliable.
- ***Aphasia assessment****: The ABC test is administered only to individuals with aphasia to describe the level of impairment. Since implementation outcomes are the primary focus, participants who are unable to complete the questionnaires independently (e.g. due to aphasia) are not required to do so. Support of proxies in completing the questionnaires is allowed, however proxies are asked not to complete the questionnaires on behalf of participants. We have added this to the manuscript on page 12-13 lines 24; 1-3. Similarly, participants with motor impairments are not required to perform the one-minute sit-to-stand or 10-meter walking tests.*
- ***Missing data****: Data will be analysed with GEE models for repeated measurements, which allows inclusion of participants with incomplete data in the analyses if the assumption of missing-at-random is met. We have added this to the manuscript on page 18, lines 12-13:*

"This method takes all observations into account, even if some outcomes are missing, assuming that these data are missing at random."

- ***Cost evaluation**: We have specified that a descriptive cost analysis will be conducted using data from the iMCQ and iPCQ questionnaires, focusing on healthcare utilization and productivity losses. A full cost-effectiveness analysis is not planned. We have added this to the manuscript on page 16, lines 14-16: "Data from the iMCQ and iPCQ questionnaires will be used to conduct a descriptive cost analysis focusing on healthcare utilization and productivity losses."*

- **Sample size estimation and statistical analyses**

As mentioned above, the power calculation (P7) is based on the premise that the researchers are looking at a simple difference in proportions of participants engaging in the sports consultancy through the SportMEE program. This is an implementation outcome. However, in the statistical plan (P17) the authors report intending to conduct a repeated measures analysis with four timepoints including the baseline. This is an efficacy outcome. The study is under powered to include the many potential covariates. The authors do not specify what specific covariates will be included in the models, nor what is the justification for their inclusion (ie. what change would they expect to see in the experimental group versus controls). It is recommended that the researchers consult a statistician for the power calculation and the analysis. At the very least, the power calculation (sample size) should be based on the analysis of the primary outcome, and this needs to be determined (what outcome measure will best detect a difference between groups and what is the MDC or MCID of that measure).

Response: We agree with the reviewer that the previous version did not sufficiently specify the primary outcome. We have clarified in the manuscript that the primary outcome of the study is the implementation outcome reach, defined as the proportion of participants engaging in the SportMEE consultancy program after discharge. The power calculation is therefore considered appropriate based on this implementation outcome and study design.

In addition, we acknowledge the reviewer's concerns regarding the repeated measures analyses with four timepoints and the inclusion of potential covariates. With four measurements per patient more than 200 observations will be available for the analyses per outcome, which will provide insight in recovery patterns over time for both groups. These analyses are exploratory, as the study was not powered to detect statistically significant effects for a specific outcome. The covariates will be selected based on theoretical or clinical relevance and the maximum number of

covariates in the model will be based on the number of participants in the study population. All results of the secondary analyses will be interpreted with caution.

- Some specific comments re wording / language are listed below for the authors consideration. Wording which could be altered is highlighted in **bold**.

Response: All rewording / language comments are changed in the manuscript using track changes. For other responses, please see below.

- P3 line 6. This dot point could be reworded …"**allows the research team** to adjust…";
- P4 line 44. .."shown **to be** effective"…
- P5 line 33 …were in line"… (not where)
- P5 line 35 …"**will** continue to be involved"…
- P5 line 42 …"independent user committee **has been**…"
- P5 line 50-51 …The committee **will meet** once a year.
- P6 line 17 …"aims to advise"…(not advice)
- P6 Line 46. A more appropriate term would be those with moderate to severe cognitive impairment (to replace mentally incapacitated).

  Response: We have added the following text to the manuscript on page 7, lines 20-22: "Individuals with a life expectancy of less than 1 year or with severe cognitive impairments that preclude providing informed consent and sufficient understanding of study procedures are excluded from the study."
- Recruitment (Page 7) How will capacity to consent be assessed?

  Response: Capacity to consent and participate is assessed by the treating rehabilitation physician, this is added to the manuscript on page 7, lines 20-22.
- P7 lines 23-26 and 46-51 include the same information.
- P8 line 32. What are the eligibility criteria for this service (eg. mild to moderate ABI)?

  Response: There are no eligibility criteria for the SportMEE consultancy program. This is added to the manuscript on page 9, lines 10-11.
- P8 line 48 Should mention be made of carers? Many individuals with ABI will need considerable support (from family/relevant others) to engage in this program.

  Response: Indeed individuals with ABI may need support to engage in this study and the SportMEE consultancy program. However, family members or other carers are not recruited for the study, since they do not complete the program or questionnaires on behalf of participants.
- Table 2, P10, line 11. …"not aware of the advantages **of an** active lifestyle"…
- P11 Data collection. Lines 12 and 14. Two sentences commence with "In addition…"

- P13 line 41. How does ‘dose delivered’ differ from ‘dose received’
  Response: Within the RE-AIM framework dose delivered refers to what is actually delivered (e.g. number of SportMEE consultations), where dose received refers to what the participants actually receives or engages in (e.g. number of consultancy sessions attended by the participants).
- P15 line 17. …”drug us**e**”…

VERSION 2 - REVIEW

Reviewer 2

Name Singer , Barbara

Affiliation School of Medical and Health Sciences, Edith Cowan University, Joondalup, Australia., School of Medical and Health Sciences

Date 25-Jun-2026

COI

The authors have addressed the reviewers’ comments adequately. Two minor points remain: (1) Should the headings for the manuscript sections align with the abstract headings? (eg. ‘Background' would become ‘Introduction'), and (2) it is conventional for in text numbers lower than ten to be written in full (eg. two rather than 2). I wish the authors all the best with their research.
